# Macaroni Sign in Takayasu Arteritis

**DOI:** 10.31138/mjr.050325.atr

**Published:** 2025-07-17

**Authors:** Harsh Jain, Sankar J, Kartik Sivasami, AVS Anil Kumar, Neeraj Kumar, Nidhi Goel, Ashish Chandwani, Vivek Vasdev

**Affiliations:** 1 Department of Clinical Immunology and Rheumatology, Army Hospital Research & Referral, New Delhi, India;; 2 Department of Nuclear Medicine, Army Hospital Research & Referral, New Delhi, India

**Keywords:** Takayasu arteritis, macaroni sign

An 18-year-old female presented with an 18-month history of limb claudication affecting all four limbs, along with weight loss, tender subcutaneous nodules, and high-grade fever. Examination revealed a systolic blood pressure difference of 20 mmHg between the arms, absent peripheral pulses in both lower limbs, bilateral common carotid artery with a bruit over the right and carotidynia on the left CCA. Laboratory tests showed significantly elevated inflammatory markers, with an erythrocyte sedimentation rate (120 mm/hour) and C-reactive protein (93 mg/L; normal <10). Ultrasonography demonstrated near-complete occlusion of the left common carotid artery at its origin, while the right CCA exhibited homogeneous, mid-echoic circumferential wall thickening (**Figure 1A–B**). Ultrasonography examination was performed using a 16 MHz hockey stick probe. This ultrasonographic finding is pathognomic of Takayasu’s arteritis (TAK) and has been designated the “macaroni sign”.^1^

**Figure 1.**
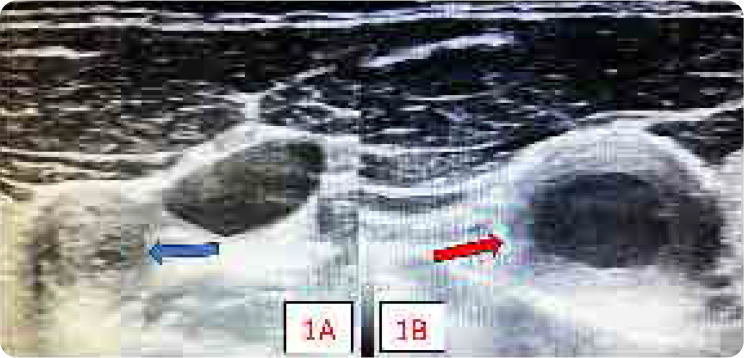
(**A**) Νear complete occlusion of left CCA (blue arrow). (**B**) Μid-echoic circumferential wall thickening of right CCA suggestive of macaroni sign (red arrow).

Positron emission tomography (PET) revealed features of type V TA along with vessel wall inflammation. Involvement of the left common carotid artery (CCA) showed long-segment narrowing approximately 4 cm from its origin with a minimal luminal diameter of 1 mm due to significant circumferential wall thickening. Beyond this segment, there was a gradual reduction in wall thickness, with an improvement in CCA calibre, reaching a maximum diameter of approximately 3.5 mm. The right CCA exhibited 50–60% luminal narrowing due to vessel wall thickening. PET findings were consistent with the ultrasonographic assessment of the CCA (**Figure 2A–B**).

**Figure 2.**
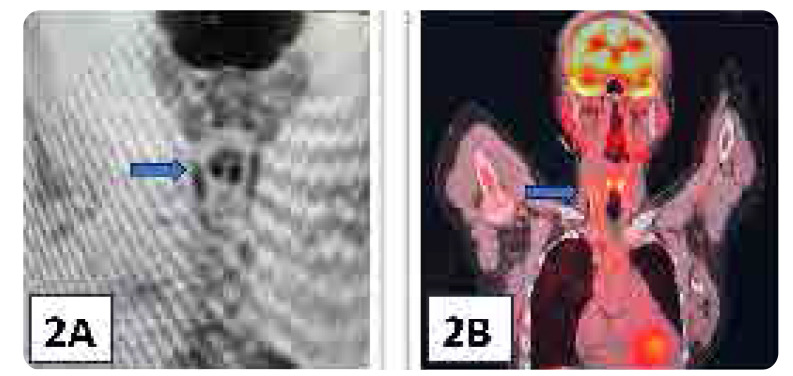
(**A, B**) Patchy FDG uptake in thickened walls of bilateral common carotid arteries with SUVmax (Maximum Standardised uptake value) of 7.2 (Blue arrows).

Takayasu arteritis is a large-vessel vasculitis of unknown cause, primarily affecting the aorta and its major branches. This leads to segmental narrowing, occlusion, or aneurysm formation. Early diagnosis is crucial, as vascular inflammation is still reversible at this stage. Delayed detection results in fibrosis of the arterial wall, leading to permanent stenosis and occlusion. Comparative research directly evaluating CDS against other imaging modalities remains limited. However, a case series by Czihal et al. demonstrated a strong agreement (83%) between CDS and 18F-fluorodeoxyglucose positron emission tomography (PET) in identifying proximal arm involvement in patients with LVV.^2^ While angiography remains the gold standard for diagnosing TAK, ultrasonography offers a non-invasive alternative, allowing for the identification of characteristic circumferential, long, and homogeneous arterial wall thickening in early disease and monitoring disease activity over time.^3^

## AUTHOR CONTRIBUTIONS

All authors made substantial contributions to the conception or design of the work, or the acquisition, analysis, or interpretation of data. All authors were involved in drafting the manuscript or revising it critically for important intellectual content, gave final approval of the version to be published, and agree to be accountable for all aspects of the work.

Harsh Jain, Sankar J, Nidhi Goel and AVS Anil Kumar contributed to data collection and analysis. Harsh Jain, Sankar J, Neeraj Kumar and S. Kartik contributed to the study design and critical revision of the manuscript. Harsh Jain, Nidhi Goel, Ashish Chandwani, and Vivek Vasdev contributed to data interpretation and manuscript preparation. All co-authors take full responsibility for the integrity and accuracy of all aspects of the work.

## PATIENT CONSENT

Written informed consent was taken from the patient.

## CONFLICT OF INTEREST

Authors declare no conflict of interest. No ethics/authorship issues related to reported images.

## FUNDING

NA.

## DATA AVAILABILITY STATEMENT

All available data is included in the manuscript.
